# Morphological Tracing and Functional Identification of Monosynaptic Connections in the Brain: A Comprehensive Guide

**DOI:** 10.1007/s12264-024-01196-0

**Published:** 2024-05-03

**Authors:** Yuanyuan Li, Yuanyuan Fang, Kaiyuan Li, Hongbin Yang, Shumin Duan, Li Sun

**Affiliations:** 1 Affiliated Mental Health Center and Hangzhou Seventh People’s Hospital and School of Brain Science and Brain Medicine, Zhejiang University School of Medicine, Hangzhou, 310058 China; 2 Liangzhu Laboratory, MOE Frontier Science Center for Brain Science and Brain-machine Integration, State Key Laboratory of Brain-machine Intelligence, Zhejiang University, Hangzhou, 311121 China; 3 NHC and CAMS Key Laboratory of Medical Neurobiology, Zhejiang University, Hangzhou, 310058 China; 4 Department of Neurobiology and Department of Neurology of the Second Affiliated Hospital, Zhejiang University School of Medicine, Hangzhou, 310058 China; 5 Institute of Basic Medical Sciences, Chinese Academy of Medical Sciences and Peking Union Medical College, Beijing, 100005 China

**Keywords:** Trans-monosynaptic, Retrograde, Anterograde, Viral tool, Neural circuit, Optogenetics, Electrophysiology

## Abstract

Behavioral studies play a crucial role in unraveling the mechanisms underlying brain function. Recent advances in optogenetics, neuronal typing and labeling, and circuit tracing have facilitated the dissection of the neural circuitry involved in various important behaviors. The identification of monosynaptic connections, both upstream and downstream of specific neurons, serves as the foundation for understanding complex neural circuits and studying behavioral mechanisms. However, the practical implementation and mechanistic understanding of monosynaptic connection tracing techniques and functional identification remain challenging, particularly for inexperienced researchers. Improper application of these methods and misinterpretation of results can impede experimental progress and lead to erroneous conclusions. In this paper, we present a comprehensive description of the principles, specific operational details, and key steps involved in tracing anterograde and retrograde monosynaptic connections. We outline the process of functionally identifying monosynaptic connections through the integration of optogenetics and electrophysiological techniques, providing practical guidance for researchers.

## Introduction

With the application of new technologies such as optogenetics [1], neural mechanisms underlying brain functions, particularly behavioral brain mechanisms, have been extensively investigated [2–5]. Decoding neural circuits has become a vital goal in the study of behavioral brain mechanisms. Because of the complexity of neural circuits, it is exceptionally challenging to analyze the entire circuitry involved in specific behaviors. Practically, before completely dissecting the complicated neural circuitry, it is necessary to first identify the upstream and downstream neurons that have direct synaptic (monosynaptic) connections with the neurons of interest [6–8]. The most commonly used method for morphologically identifying monosynaptic connections is the recently developed trans-synaptic viral tracing technique, which includes retrograde [9–14] tracing systems to identify upstream neurons and anterograde [15–18] tracing systems to identify downstream neurons. With the use of optogenetics to specifically stimulate the terminals of neurons of interest projecting to specific target areas and simultaneously recording postsynaptic potentials in the target area neurons, combined with pharmacological analysis, it is feasible to functionally identify whether there are direct monosynaptic connections between two types of neurons or whether there are polysynaptic connections mediated by other neurons [4, 19–21]. Together with the pharmacological approach, it is also feasible to determine whether the connection is excitatory glutamatergic or inhibitory GABAergic [2, 5, 22, 23].

**Fig. 1:**
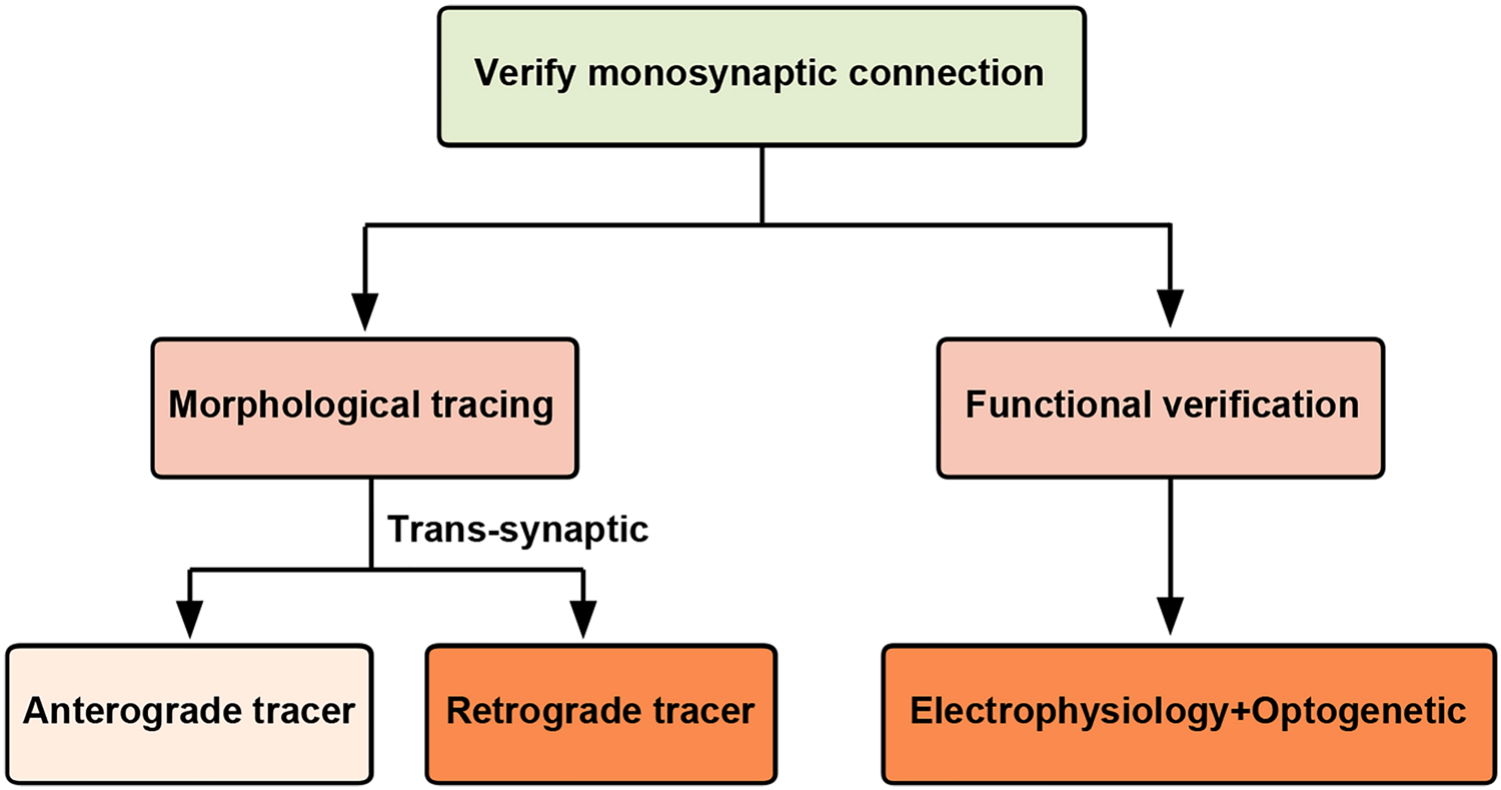
Schematic summary for identification of the monosynaptic innervation. The morphological identification of monosynaptic connections is achieved by employing viral tracing. The functional identification of monosynaptic connections involves the integration of optogenetics with electrophysiological recordings and pharmacological methods.

In this paper, on the basis of the literature reported and the experience accumulated in our laboratory, we provide a comprehensive description of the morphological identification of monosynaptic connections using viral tracing and the functional identification of monosynaptic connections using optogenetics combined with electrophysiological recordings and pharmacological methods (Fig. 1). We also highlight important technical considerations and potential issues in the experimental procedures for the convenience and feasibility of the techniques.

## Viral Tracing Strategy

### Anterograde

#### Herpes Simplex Virus

Herpes simplex virus (HSV) is a double-stranded DNA virus with an anterograde trans-synaptic transmission function. Zeng *et al.* [15] successfully engineered a modified form of the H129 strain of HSV1, known as H129-ΔTK-tdT, through the deletion of the thymidine kinase (TK) gene from its genetic makeup. This modification enabled the virus to selectively cross a single synapse. However, the use of this HSV variant still necessitates the presence of a helper virus that expresses TK. With the use of AAV-TK-GFP, anterograde tracing has been achieved in wild-type mice, whereas AAV-DIO-TK-GFP facilitates the specific labeling of downstream neurons in mice expressing Cre recombinase in specific types of neurons. Recently, Yang *et al.* [16] created H129-dTK-T2, an updated version of H129-dTK-tdT, which has improved labeling intensity. However, the pronounced neurotoxicity of HSV poses considerable limitations on its utility for the long-term and precise mapping of neural circuits [24–26].

#### Recombinant Adeno-associated Virus

Recombinant adeno-associated virus (rAAV) was derived as a gene transfer vector from the wild-type AAV, a member of the *Parvoviridae* family and the *Dependovirus* genus. A diverse range of 13 serotypes [24] (AAV1–13) and > 100 variants have been identified across various host species. AAV1, AAV2, AAV5, AAV8, and AAV9 are among the commonly used serotypes in neural circuits [25]. Among them, AAV1 and AAV9 have been reported to have the ability to label neurons anterogradely across synapses [17]. It should be stated that in comparison with HSV, AAV1 may have a lower probability of retrogradely labeling neurons. However, rAAV is capable of achieving the long-term and stable expression of foreign genes *in vivo* primarily because of its low immunogenicity, diverse serotypes, and broad host cell tropism.

Synaptophysin is a membrane protein predominantly located within the synaptic vesicles of neurons and serves as a reliable marker for synaptic terminals [18, 27]. It is commonly used in immunohistochemical studies to visualize [18, 28, 29] and quantify [4] synaptic terminals in brain tissue sections, facilitating investigations into synaptic connectivity, development, and activity. It can be used to differentiate transit fibers from actual synaptic terminals. Synaptophysin is commonly delivered using AAV vectors, which can be engineered with various promoters to achieve expression in wild-type mice or in a Cre-dependent manner.

### Retrograde

#### Rabies Virus

The wild-type rabies virus (RV) is an RNA virus that spreads well between synaptically connected neurons in a retrograde manner. However, its broad infectivity prevents its direct use for monosynaptic tracking [11, 12]. To selectively label neurons that possess monosynaptic connections to specific cell types, researchers developed a modified RV system that relies on the synergistic action of Cre recombinase and helper virus [9, 11, 12, 30–34]. Glycoprotein promotes the fusion of virus and host cell membranes and provides internalized virus particles with intracellular transport characteristics, infecting presynaptic terminals to bring the retrograde trans-synaptic transmission of the RV into effect. RV-ΔG pseudotyped with EnvA, the avian sarcoma, and leukosis virus A protein, cannot infect mammalian neurons. It can only infect cells that are specially engineered to express the avian cell-surface molecule tumor virus A (TVA), the receptor of EnvA. This receptor-ligand pair (TVA-EnvA) allows specific infection of target neurons. RV-ΔG is replication-deficient, so it cannot spread beyond the initially infected neurons unless the glycoprotein is supplied. With the adeno-associated helper virus that expresses TVA and RVG, the virus can produce infectious progeny, which spread from these neurons to the presynaptic neurons directly connected to them [24, 30, 35, 36].

#### Canine Adenovirus Type 2

Canine adenovirus type 2 (CAV-2) exhibits high efficiency in transducing axon terminals and undergoes retrograde transport to reach the cell bodies of upstream neurons [37]. CAV-2 stands out because it relies on a single, highly conserved cell adhesion molecule known as the Coxsackie virus and adenovirus receptor [38]. CAV-2 is commonly engineered to express Cre recombinase, which is subsequently injected into a specific region. Following this, a Cre-dependent AAV is administered into the targeted upstream nucleus. CAV-cre is transported retrogradely from the distal axon terminals to the cell body, facilitating the expression of Cre recombinase in the nucleus. Consequently, the target gene is selectively activated in a subset of neurons that project from the source to the target area [25, 39].

#### rAAV2-retro

rAAV2-retro is a genetically engineered version of AAV2 that has undergone a selection process driven by evolution to enhance its effectiveness in retrograde axonal transport. It is efficiently captured by axon terminals and undergoes retrograde transport to reach the cell bodies in remote brain regions, where transgene expression can be observed [40]. The rAAV2-retro system can be used independently or in combination with Cre recombinase driver lines to establish sustained and robust transgene expression, facilitating the efficient exploration of neural circuit function [14]. However, rAAV2-retro does not appear to work well in the viral tracing involved in GABAergic neurons in our practical applications.

During the last decade, immense changes have transpired in neural circuit research owing to the rapid development of viral tools. Here, we summarize viruses commonly used for neural circuits (Table 1).

**Table 1:** Popular viral tracing strategies for neural circuit.

Type	Virus	Commonly used viral vectors	Helper virus/mice	Recommended concentration	Expression time	Trans-monosynaptic
Anterograde	HSV	H129△TK-ubc-tdTomato	AAV-TK	≧1.00 × 10^9^ PFU/mL	2–5 days	Yes
		HSV-△TK-LSL-tdTomato	AAV-DIO-TK	≧1.00 × 10^9^ PFU/mL	2–5 days	Yes
	AAV	AAV1-cre	Cre reporter mouse or Cre-dependent virus	≧1.00 × 10^12^ vg/mL	3 weeks	Yes
		AAV-FLEx-EGFP-2A- Synaptophysin-mRuby	–	≧1.00 × 10^12^ PFU/mL	3 weeks	No
Retrograde	RV	RV-ΔG-EnVA	Mixture (1:1) TVA and RVG (Cre-dependent or not)	≧2.00 × 10^8^ IFU/mL	3 weeks for helper + 1 week for RV	Yes
	CAV	CAV2-cre	Cre reporter mouse or Cre-dependent virus	≧4.00 × 10^12^ vg/mL	1–2 weeks	No
	rAAV2-retro	AAV2/2-EF1α-DIO-EYFP	–	≧1.00 × 10^12^ vg/mL	3 weeks	No
		AAV2/2-hsyn-tdTomato	–	≧1.00 × 10^12^ vg/mL	3 weeks	No
		AAV2/2-hsyn-cre	Cre reporter mouse or Cre-dependent virus	≧1.00 × 10^12^ vg/mL	3 weeks	No

The biosafety level of laboratory when applying tracing virus is ABSL-2 (P2) qualified.

### Electrophysiology Combined with Optogenetics

The patch-clamp technique is currently the most common technology used to study the action potential (AP) and neural activity [41–43]. Optogenetics can be used to visualize neurons of the central nervous system and achieve remote control of animal behaviors through the photostimulation of particular types of neurons [1, 44, 45]. Boyden *et al.* first established a method combining *in vivo* electrophysiology and optogenetics to precisely control the excitability and excitatory or inhibitory synapses of specific neurons [19].

**Fig. 2:**
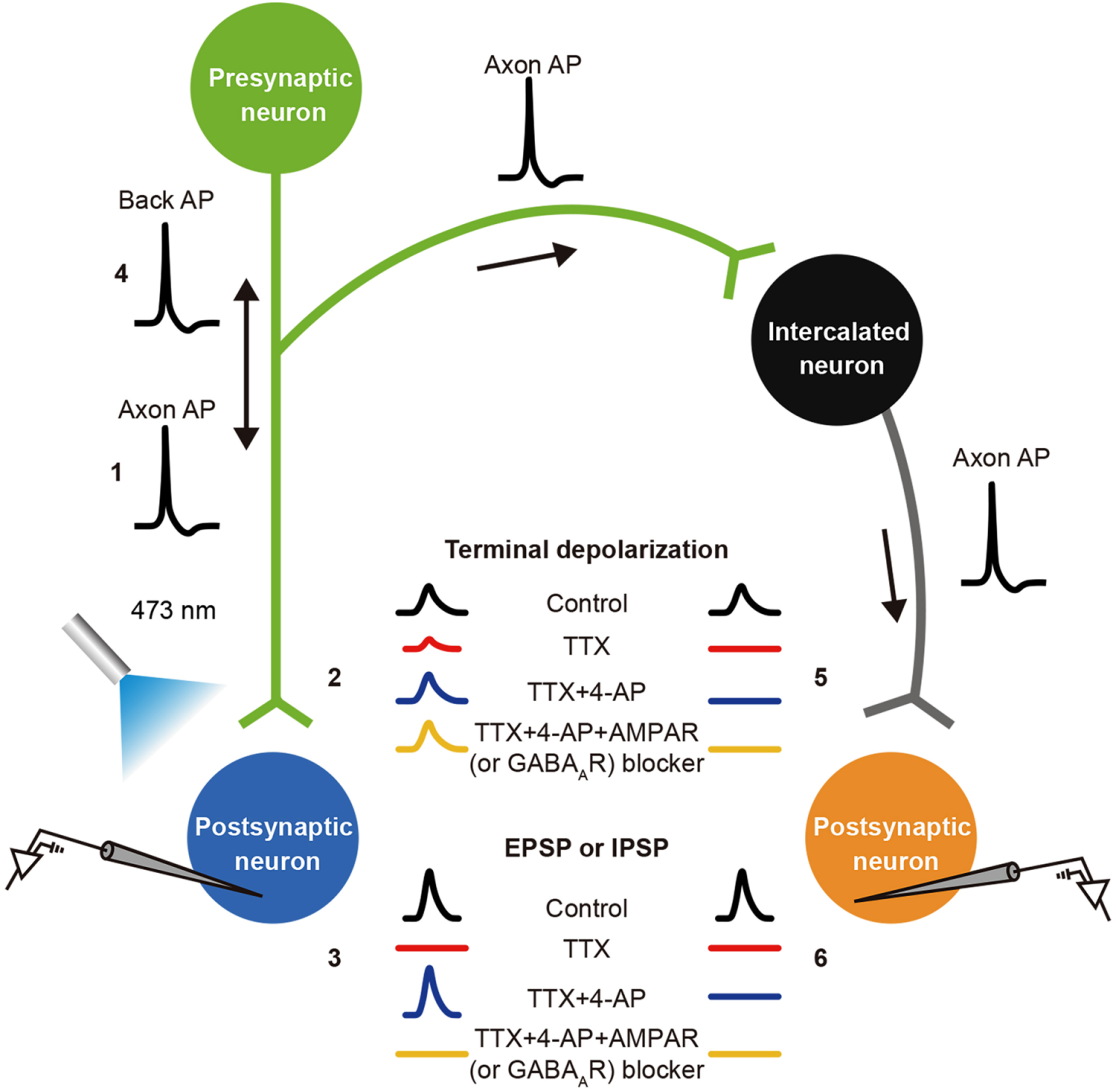
Functional identification of monosynaptic connections by optogenetics combined with electrophysiology and pharmacology. Optogenetic stimulation (blue light, 473 nm) in the downstream target area of axon terminals of neurons (green) expressing ChR2 evokes the “all-or-none” AP (1), which induces significant depolarization (2, black trace), activating voltage-gated Ca^2+^ channels in the terminals and triggering synaptic vesicle release, resulting in a postsynaptic potential in the monosynaptic connected neurons (3, black trace). This AP can also propagate retrogradely (backpropagation) along the axon toward the cell body (4) and spread through axon collaterals, triggering excitation in the intercalated neurons (black), which induces AP-driven terminal depolarization (5, black trace) and postsynaptic potential (6, black trace) in the polysynaptically connected neurons (yellow). Application of the Na^+^ channel blocker TTX can block the generation and propagation of APs induced by optogenetic stimulation, thereby blocking AP-driven terminal depolarization and the postsynaptic potentials in both monosynaptically (2 and 3, red traces) and polysynaptically connected neurons (5 and 6, red traces). Although optogenetic stimulation can induce small depolarization in ChR2-expressing neural terminals (2, red trace), this depolarization is insufficient to induce voltage-gated Ca^2+^ channel activation and synaptic vesicle release. Application of 4-AP increases membrane depolarization, membrane resistance, and Ca^2+^ channel activation, which amplifies the optogenetic-induced Ca^2+^ influx in ChR2-expressing neural terminals (2, blue trace) and triggers synaptic vesicle release, thereby inducing postsynaptic potentials in monosynaptically connected neurons (3, blue trace). However, in the polysynaptically connected neurons, postsynaptic potentials cannot be recorded (6, blue trace) because of a lack of direct light-induced neural terminal depolarization. Pharmacological approaches can further identify the neurotransmitter type involved in synaptic transmission, which in the brain is primarily excitatory glutamatergic synapses and inhibitory GABAergic synapses (3, yellow trace).

The adeno-associated virus expressing Channelrhodopsin-2 (ChR2) is first injected into the upstream nucleus, then the downstream terminals are photostimulated, and the postsynaptic potentials produced on the cells surrounded by the terminals are recorded using the whole-cell patch-clamp technique [1]. The application of tetrodotoxin (TTX; 1 μM) blocks voltage-gated Na^+^ channels, the generation or conduction of AP is prevented, and the AP-dependent terminal depolarization and synaptic transmission are eliminated [46] (Fig. 2). Thus, light stimulation fails to induce APs and postsynaptic potentials in both monosynaptic and polysynaptic connected neurons in the presence of TTX (Fig. 2). The application of 4-aminopyridine (4-AP; 100 μM) not only blocks K^+^ channels that induce membrane depolarization and increase the membrane resistance but also directly facilitates Ca^2+^ channels [47], thus amplifying the ChR2-mediated depolarization of presynaptic terminals and presynaptic Ca^2+^ channel activation. The postsynaptic responses (excitatory or inhibitory postsynaptic potential) in monosynaptically innervated neurons are thus recorded again even if in the presence of TTX (Fig. 2). Simply put, in the presence of 4-AP, TTX can only block AP-induced terminal depolarization and synaptic transmission in polysynaptically connected neurons but not the light-induced depolarization of ChR2-expressing terminals and synaptic transmission in monosynaptically connected neurons (Fig. 2). In addition, the application of specific receptor antagonists helps to verify the type of the transmitter involved in the monosynaptic connection [41, 42]. The application of a glutamate receptor antagonist effectively blocks the excitatory postsynaptic current, providing evidence that the monosynaptic connection is mediated by glutamatergic transmission. Glutamate antagonists are primarily classified into two categories: non-*N*-methyl-d-aspartate receptor antagonists (including 6-nitro-7-sulfamoylbenzo(*f*)-quinoxaline-2,3-dione, 6-cyano-7-nitroquinoxaline-2,3-dione (CNQX) and 6,7-dinitroquinoxaline-2,3-dione), *N*-methyl-d-aspartate receptor antagonists (such as d-2-amino-5-phosphonovaleric acid and dizocilpine). By applying a GABA receptor antagonist, the inhibitory postsynaptic current is effectively blocked, presenting compelling evidence that the monosynaptic connection operates through GABAergic transmission. The GABA_A_ receptors serve as the binding sites for commonly applied GABA receptor antagonists in electrophysiology, including bicuculline, picrotoxin, and GABAzine (4,5,6,7-tetrahydroisoxazolo[4,5-*c*]pyridin-3-ol) [2–4, 29, 48–50].

The light-induced responses of monosynaptically and polysynaptically innervated neurons under different treatments are also listed in Table 2.

**Table 2:** Light-induced responses in mono- and polysynaptic innervated neurons under various treatments.

Treatment	Axon AP (1)	Back AP (4)	Terminal depolarization		EPSP or IPSP	
			Monosynaptic (2)	Polysynaptic (5)	Monosynaptic (3)	Polysynaptic (6)
Control	Yes	Yes	Large	Large	Yes	Yes
TTX	No	No	Small	No	No	No
TTX+4-AP	No	No	Large	No	Yes	No
TTX+4-AP+AMPAR (or GABA_A_R) blocker	No	No	Large	No	No	No

## Procedures

### ***First Strategy: Monosynaptic Retrograde Viral Tracing in Adult Mice (***Fig. 3***)***

**Fig. 3:**
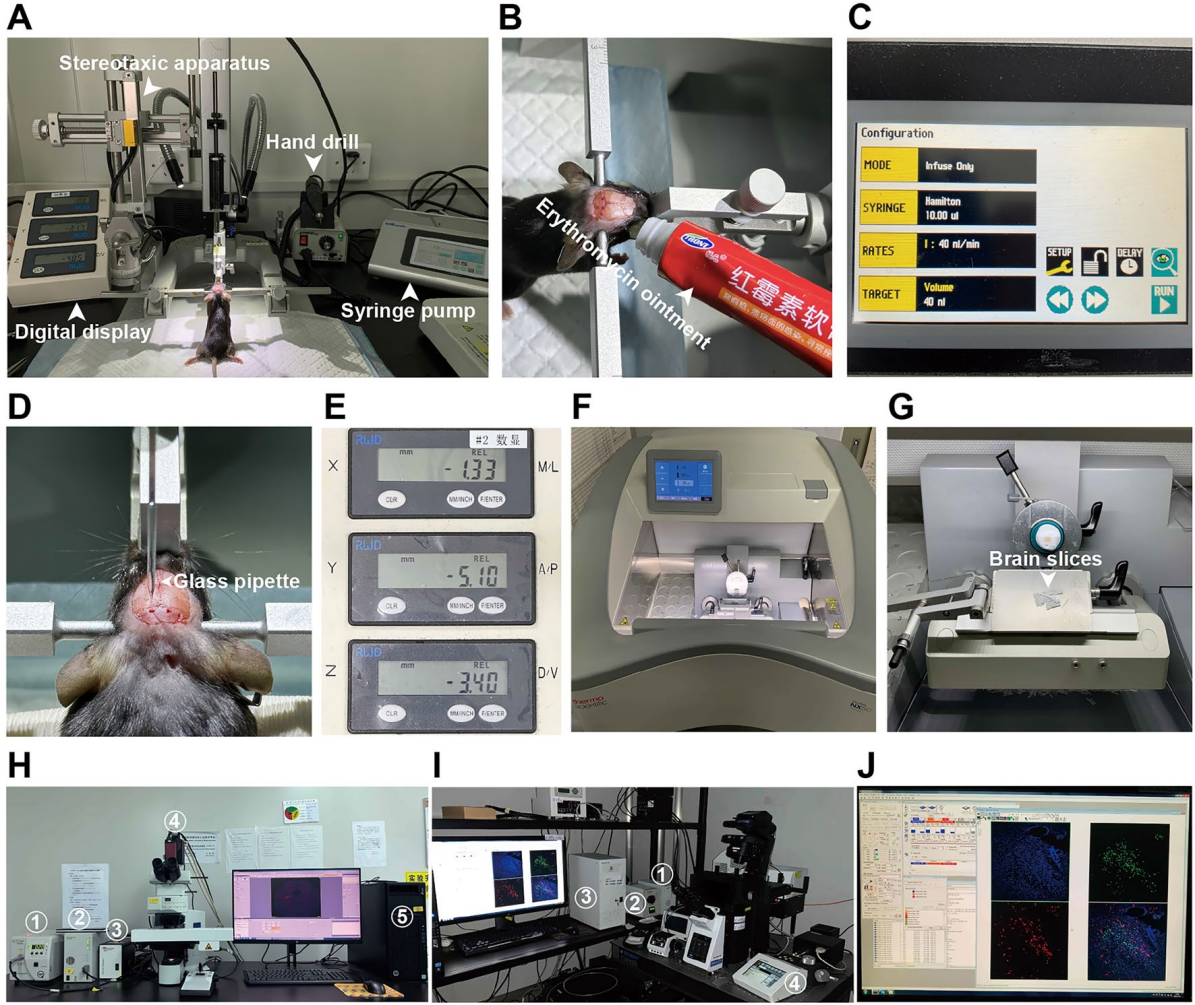
Equipment required for stereotaxic injections and microscopic imaging in mouse brain. A Equipment setup for the virus injection. B Fix the mouse on the stereo apparatus, expose the skull by incising the scalp, and apply erythromycin ointment on both eyes. C The parameter setup of virus injection on the syringe pump (KD Scientific). D Inject the virus using a glass pipette (Sutter Instrument). E The coordinates of the LPB. F and G Slice the brain using CryoStar NX50 OP cryostat (Thermo). H Fast large-scale imaging system for examining the distribution pattern of virus-infected neurons across the whole brain using the virtual digital slide slice scanning system VS120 (Olympus) (1, mercury lamp; 2, microscope controller; 3, electric stage controller; 4, 16-bit sCMOS camera; and 5, computer). I High-magnification imaging system for the comprehensive examination of the distribution of virus-infected neurons and their axon terminals in the local brain area using the laser scanning confocal microscope FV-1200 (Olympus) (1, microscope power supply system; 2, mercury lamp; 3, main controller; and 4, microscope touch operating system). J FV10-ASW application software.

*Step 1* *:* *Viral Stereotaxic Intracerebral Injections*

! *CAUTION* Make sure that the biosafety level of the laboratory when applying tracing virus is at least ABSL-2 (P2).

- *X*-Cre mice aged 8–10 weeks are prepared for surgery.
- Each mouse is anesthetized and positioned in the stereotactic frame.
- All stereotaxic surgeries are performed under pentobarbital sodium (0.1 g/kg i.p.) anesthesia (Fig. 3A).
- Each mouse’s head is fixed on a stereotaxic apparatus (RWD, Shenzhen, China) (Fig. 3D).
- Erythromycin ointment is applied to maintain eye lubrication (Fig. 3B).
- The mouse’s head is aligned horizontally, ensuring that the height difference between the front and rear fontanels is minimal, ideally < 0.05 mm.
- The location of the lateral parabrachial nucleus (LPB, bregma, 5.10 mm; lateral, ±1.33 mm; ventral, 3.40 mm) is verified using the stereotaxic apparatus (RWD) under the binocular stereotaxic microscope (Olympus, Tokyo, Japan).
- A manual drill (Saeshin Precision, Daegu, Korea) with a round tip drill bit (Hager & Meisinger GmbH, Centennial, CO, USA) is used to open the skull, exposing the brain surface above the target area.
- A saline-soaked cotton pad is used to wipe the skull, maintaining its cleanliness and moisture.
- For monosynaptic retrograde tracing, 60–80 nL of a mixture (1:1) of AAV2/9-rAAV-EF1α-DIO-oRVG-WPRE-hGH pA (5.40 × 10^12^ genomic copies per mL, BrainVTA, Wuhan, China) and AAV2/9-rAAV-EF1α-DIO-H2B-EGFP-T2A-TVA-WPRE-hGH pA (5.24 × 10^12^ genomic copies per mL, BrainVTA) is unilaterally injected into the LPB at 40 nL/min using a single syringe infuse/withdraw system (KD Scientific, Holliston, MA, USA) connected to a glass pipette (tip diameter 10–30 μm, Sutter Instrument, Novato, CA, USA) (Fig. 3 C–E).

▲*CRITICAL STEP* The pipette is withdrawn 10–15 min after the end of the infusion. The incision is closed with a suture and a tissue adhesive (Vetbond; 3M, St Paul, MN, USA).

! *CAUTION* Make sure to sterilize the scalp with iodophor to avoid bacterial infection.

- Three weeks later, 80–100 nL of EnvA-pseudotyped, glycoprotein (G)-deleted and DsRed-expressing RV (RV-EnVA-ΔG-DsRed) (5.0 × 10^8^ genomic copies per mL, BrainVTA) is injected into the same site of LPB.

▲*CRITICAL STEP* The injection needle is withdrawn 10–15 min after the end of the infusion and the incision is closed with a suture and a tissue adhesive (Vetbond; 3M).

*Step 2: Perfused Fixation*

- One week after injection of RV-EnVA-ΔG-DsRed, mice were deeply anesthetized and transcardially perfused with 0.1 mol/L of PBS and 4% paraformaldehyde in PBS.
- Their brains are then removed and fixed in 4% paraformaldehyde buffer at 4°C overnight. After fixation, the brains are cryoprotected in 30% sucrose (wt/vol) at 4°C for at least 48 hours to ensure complete dehydration.

▲*CRITICAL STEP* When the brain has completely sunk to the bottom of the 30% sucrose solution, it can be determined that the brain is adequately dehydrated and ready for sectioning. Otherwise, there is a risk of damaging the brain tissue during the frozen section procedure.

*Step 3: Serial Frozen Sections*

- Brains frozen in optimal cutting temperature compound (Sakura, Torrance, CA, USA) were sectioned at 40 μm in the coronal plane using the CryoStar NX50 OP cryostat (Thermo, Waltham, MA, USA) (Fig. 3F, G).

*Step 4: Immunohistochemistry*

- The brain slices are rinsed three times with 0.1 mol/L of PBS for 5 min each.
- The samples are incubated in DAPI (Beyotime, Shanghai, China) for 2 min.
- The samples are sealed with a mounting medium (90% glycerol in 0.1 mol/L PBS).

! *CAUTION* Do not shake the mounting medium, and make sure there are no bubbles within the mounting medium. If there are bubbles in it, place the medium in the fridge and let it sit for 1 h before use.

*Step 5: Microscopic Imaging*

- Brain sections are imaged using a virtual digital slide slice scanning system VS120 (Olympus) (Fig. 3H) for whole-brain screening of the regions that are labeled with rabies. Then, each rabies-labeled nucleus was imaged at high resolution using a laser scanning confocal microscope FV-1200 (Olympus) for individual cell morphology analysis and quantitative statistics (Fig. 3I, J).

### ***Second Strategy: A Combination of Electrophysiology and Optogenetics to Identify Functional Monosynaptic Connections in Acute Brain Slices (***Fig. 4***)***

**Fig. 4:**
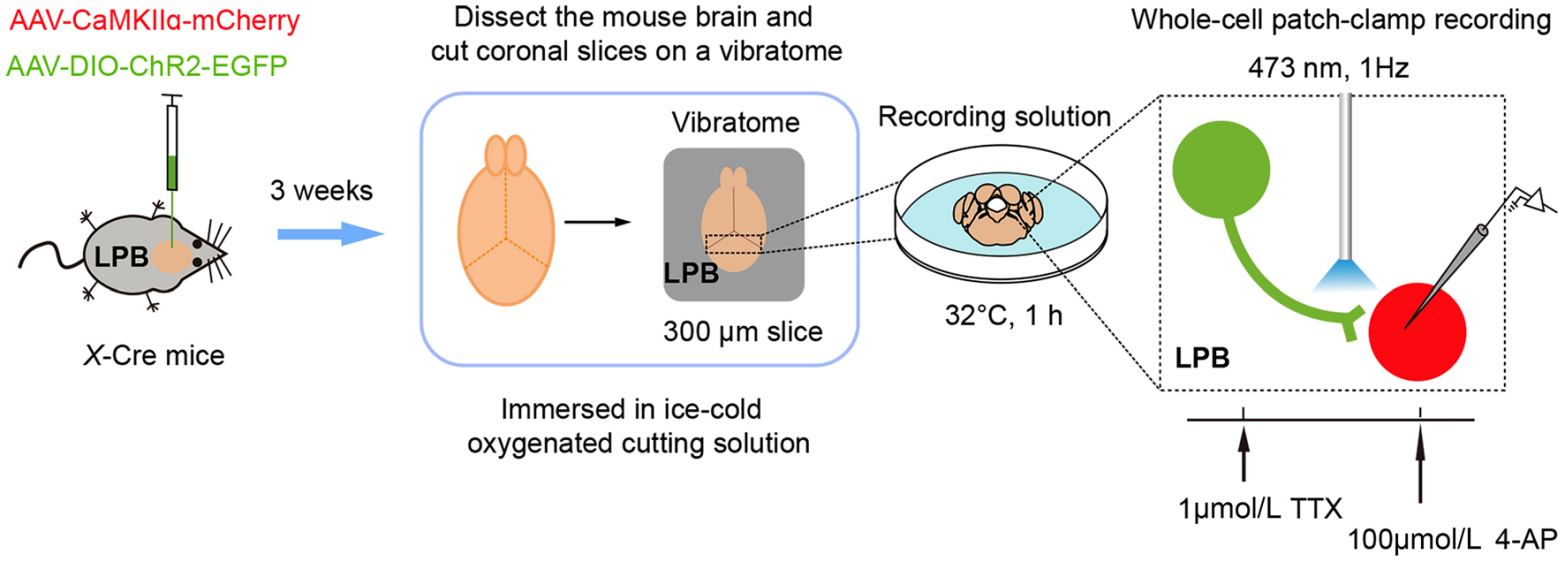
Schematic of functional identification of monosynaptic connection by electrophysiology combined with optogenetics and pharmacological approaches using the brain slice preparation. The AAV-CaMKIIα-mCherry and AAV-DIO-ChR2-EGFP viruses were injected into the LPB of *X*-Cre mice for 3 weeks, each mouse was anesthetized with isoflurane, and their brains were dissected rapidly, and immersed in ice-cold oxygenated (95% O_2_/5% CO_2_) cutting solution. Coronal slices (300 μm, magnified view of the first dashed area LPB) were cut on a vibratome and then allowed to recover in the recording solution. Neurons, axons, and synaptic terminals expressing ChR2 are activated by 10 ms flashes of blue light (473 nm, 1 Hz) from a single-wavelength laser system. To confirm that postsynaptic currents are monosynaptic, EPSCs (recorded at −50 mV membrane potential) or IPSCs (recorded at 0 mV membrane potential) were recorded in ACSF containing TTX (1 μmol/L), followed by a combination of TTX and 4-AP (100 μmol/L). The second dashed area is the magnified view of the electrophysiology process in the LPB from coronal slices. Refer also to Fig. 2.

*Step 1* *:* *Viral Stereotaxic Intracerebral Injections*

! *CAUTION* Make sure that the biosafety level of the laboratory when applying tracing virus is at least ABSL-2 (P2).

- *X*-Cre mice aged 8–10 weeks are prepared for surgery.
- The mice are anesthetized and positioned in the stereotactic frame.
- All stereotaxic surgeries are performed under pentobarbital sodium (0.1 g/kg i.p.) anesthesia (Fig. 3A).
- The mice’s heads are fixed on a stereotaxic apparatus (RWD) (Fig. 3D).
- Erythromycin ointment is applied to maintain eye lubrication (Fig. 3B).
- The mouse’s head is aligned horizontally, ensuring that the height difference between the front and rear fontanels is minimal, ideally < 0.05 mm.
- The location of the LPB is verified using the stereotaxic apparatus (RWD) under the binocular stereotaxic microscope (Olympus).
- A manual drill (Saeshin Precision) with a round tip drill bit (Hager & Meisinger GmbH) is used to open the skull, exposing the brain surface above the target area.
- A saline-soaked cotton pad is used to wipe the skull, maintaining its cleanliness and moisture.
- 120 nL of a mixture (working titer at ~ 2–5 × 10^12^ genomic copies per mL) of pAAV2/9-CaMKIIα-mCherry (2.13 × 10^12^ genomic copies per mL original, SunBio, Shanghai, China) and AAV2/9-hEF1α-DIO-hChR2(H134R)-EYFP-WPRE-pA (1.72 × 10^13^ genomic copies per mL original, Taitool Bioscience, Shanghai, China) was unilaterally injected into the LPB at 40 nL/min using a single syringe infuse/withdraw system (KD Scientific) connected to a glass pipette (tip diameter 10–30 μm, Sutter Instrument) (Fig. 3C–E).

▲*CRITICAL STEP* The pipette is withdrawn 10–15 min after the end of the infusion. The incision is closed with a suture and a tissue adhesive (Vetbond; 3M).

! *CAUTION* Make sure to sterilize the scalp with iodophor to avoid bacterial infection.

*Step 2: Sacrifice and Sampling*

- Two weeks after the virus injection, mice are deeply anesthetized with pentobarbital sodium (0.1 g/kg i.p.).

! *CAUTION* Follow appropriate guidelines and regulations for animal experiments.

- Mice are intracardially perfused with ice-cold artificial cerebrospinal fluid (ACSF, cutting solution). Then the brain is rapidly dissected and immersed in ice-cold oxygenated (95% O_2_/5% CO_2_) cutting solution.

▲*CRITICAL STEP* This step must be executed swiftly to ensure the viability of the tissue, ideally in < 2 min.

! *CAUTION* Be careful when extracting brain tissue; do not compromise the organizational integrity.

*Step 3: Brain Slices Preparation*

- The brain tissue is trimmed and affixed to the microtome base using super glue.

! *CAUTION* It is essential to avoid applying excessive mechanical force to the brain tissue as this could lead to damage.

- Coronal slices of the LPB (300 μm) were cut on a vibrating blade microtome (Leica, Wetzlar, Germany).

▲*CRITICAL STEP* It is recommended to maintain a slicing speed of 0.08 mm/s, avoiding extremes of too fast or too slow, with an amplitude set of ~ 1.8 mm.

- Slices are transferred to a small baker with an oxygenated recording solution and the slices are allowed to recover in the recording solution for at least 1 h at 32°C.

▲*CRITICAL STEP* Ensure there are no air bubbles while oxygenating the slices; the presence of bubbles can cause the slices to float, potentially leading to cell damage.

! *CAUTION* All solutions should be oxygenated with 95% O_2_/5% CO_2_ at 0–4°C (ice-liquid mixture).

*Step 4: Recording*

- Slices are transferred to a recording chamber on an antivibration table (TMC, Peabody, MA, USA) and perfused with an oxygenated recording solution.
- A constant flow of recording solution at a rate of 2–4 mL/min is maintained using a peristaltic pump (Longer Pump, Hebei, China), and the temperature of the recording solution is controlled at ~ 30°C using an automatic temperature controller (Warner, Holliston, MA, USA).
- LPB neurons expressing mCherry also surrounded by ChR2-EGFP axon terminals are visualized under a microscope (a 40× water-immersion objective on an upright fluorescent microscope, Olympus) with a high-performance CCD Camera (COHU, Poway, CA, USA) and a black white monitor (Sunspo, Guangdong, China).
- Borosilicate glass pipettes are prepared using a horizontal pipette puller (Sutter Instrument).

▲*CRITICAL STEP* Keep the resistance within 3–5 MΩ.

- The pipette is filled with the corresponding internal solutions for light-evoked inhibitory postsynaptic currents (IPSCs) or light-evoked excitatory postsynaptic currents (EPSCs) (See the “Material, Reagents and Equipment” for details).

! *CAUTION* For IPSCs recording, 20 μmol/L CNQX and 50 μmol/L D-AP5 are added to the recording solution to block AMPA and NMDA receptors, respectively. For EPSCs recording, a total of 100 μmol/L picrotoxin is added to the recording solution to block inhibitory currents mediated by GABA_A_ receptors.

- The pipette is gently moved over the targeted cell by a Sutter MPC-200 micromanipulator (Sutter Instruments).
- A small positive pressure (about 0.3 mL air) was applied to the pipette by a 1 mL syringe.
- After obtaining a whole-cell recording, ChR2 is stimulated by flashing (5 ms) 473 nm light through the light path of the microscope using an ultrahigh-powered light-emitting diode (LED, Excelitas Technologies, Waltham, MA, USA) powered (5 mW/mm^2^) by a LED driver under computer control.
- Light-evoked GABA_A_ responses or AMPA responses are obtained every 10 s with one pulse of 473 nm light with neurons voltage clamped at 0 mV or −50 mV. Signals are amplified with Multiclamp 700A (Molecular Devices, filtered at 2 kHz, San Jose, CA, USA), digitized with Digidata 1440 A (Molecular Devices, 5 kHz), and recorded using pClamp 10.4 software (Molecular Devices) and the Mini Analysis program (Synaptosoft).
- Identifying monosynaptic inputs:

TTX (1 μmol/L, Hello Bio, Meath, Ireland) is added to the ACSF using the micromanipulator (Sutter Instrument) and the signals are recorded.

! *CAUTION* TTX is a highly potent neurotoxin and should be handled with utmost care.

4-AP (100 μmol/L) is added to the ACSF and the signals are recorded.

! *CAUTION* 4-AP is a potent neurotoxin and should be handled with utmost care.

## Results

**Fig. 5:**
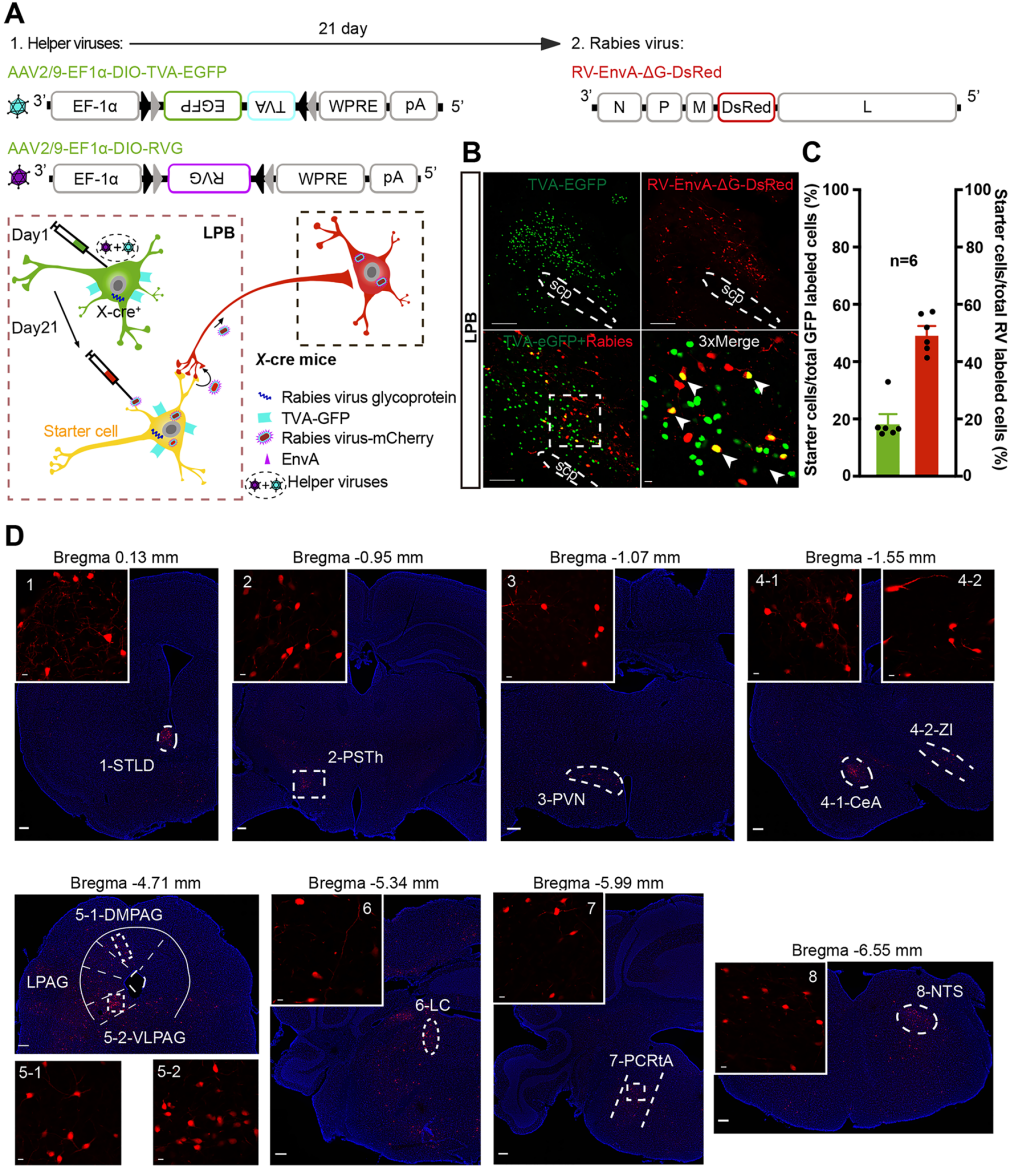
Whole-brain mapping of monosynaptic inputs to *X*-type neurons in the LPB. **A** Schematic of AAV helper virus (DIO-TVA and DIO-RVG) and rabies virus (EnvA-RV-DsRed) injections into the LPB of *X*-Cre mice. **B** Representative images of monosynaptic rabies spread from X^+^ LPB neurons in *X*-Cre mice. Arrowheads indicate starter neurons infected by both TVA-EGFP (green) and rabies virus (red). Magnified views of the boxed areas. Scale bars, 200 μm, 100 μm, and 10 μm (magnified views). **C** Percentage of starter cells that co-express with total GFP labeled cells or total RV labeled cells in the LPB (*n* = 6 sections from 3 mice). **D** Representative images of retrogradely labeled neurons in the LPB of *X*-Cre mice, receiving direct monosynaptic inputs from the STLD, PSTH, PVN, CEA, ZI, DMPAG, VLPAG, LC, PCRtA, and NTS (areas shown by dashed lines). STLD, bed nucleus of the stria terminal, lateral division, dorsal part; PVN, paraventricular hypothalamic nucleus; CeA, central amygdaloid nucleus; ZI, zona incerta; PSTh, parasubthalamic nucleus; DMPAG, dorsomedial periaqueductal gray; VLPAG, ventrolateral periaqueductal gray; LC, locus coeruleus; PCRtA, parvicellular reticular nucleus, alpha part; and NTS, nucleus of the solitary tract. Scale bars, 200 μm and 10 μm (magnified views).

Using the G-deleted RV-based tracing strategy, we injected helper viruses AAV2/9-EF1α-DIO-RVG and AAV2/9-EF1α-DIO-TVA-EGFP on day 1 and RV-EnVA-ΔG-DsRed on day 21 into the LPB of *X*-Cre mice. On day 28, we observed the localization of starter cells (indicated in yellow) in the LPB nucleus and retrogradely labeled neurons in multiple regions, which suggests that X^+^ LPB neurons directly receive monosynaptic inputs from the STLD, PSTH, PVN, CEA, ZI, DMPAG, VLPAG, LC, PCRtA, and NTS (Fig. 5).

## Discussion

We outlined two distinct, streamlined, and readily reproducible protocols for monosynaptic neuronal tracing in adult mice. By harnessing the power of versatile recombinases, viral tools, and electrophysiology, researchers can selectively label subpopulations of specific neurons. These approaches significantly facilitate investigations into the interactions between different brain regions, encompassing a broad spectrum of trans-synaptic neural circuit tracing. These methods are designed to expedite the mastery of these experimental techniques for any researcher.

The implementation of a viral tracing strategy renders the validation of single-synapse tracing in terms of morphology attainable and robust. The adapted RV system enables the selective labeling of a single neuron type across species, facilitating the search for upstream nuclei with monosynaptic connections. The synergistic use of helper viruses and transgenic mice serves to effectively restrict G and TVA expression to specific cells in particular locations, minimizing G expression in other neurons and the potential for trans-synaptic viral spread. This comprehensive approach yields unparalleled insights into the neural circuit mechanisms governing behavioral encoding and the formation of functional brain maps. Nevertheless, the current viral strategy still has limitations in various aspects, including, but not limited to, their capacity to label only a portion of input cells, resulting in variable labeling efficiency of these cells. Despite the retrograde propagation of RV exclusively between connected central nervous system neurons, anterograde labeling may occur in peripheral sensory neurons. The adapted rabies virus retains a certain level of toxicity, and prolonged expression can lead to neuronal death. Nonetheless, another drawback lies in necessitating a second craniotomy, a procedure with slight risks that demands a high level of precision during surgery. The post-initial craniotomy suturing is especially crucial because suboptimal recovery and the development of skull lesions can compromise the accuracy of the subsequent craniotomy [12].

Moreover, patch-clamp techniques offer an additional electrophysiological dimension for confirming single-synapse tracing. Recent findings lend strong support to the idea that combining electrophysiology with optogenetics furnishes unequivocal proof of single-synapse tracing, now considered the gold standard for validating neuronal functional connectivity [19, 51]. Electrophysiological methods empower researchers to delve into neuronal connections and distributions in diverse animal models with enhanced temporal and spatial precision. Experimenters gain greater control over experimental parameters like voltage, current, and other stimulation modes, enabling long-term recordings of single cell membrane potentials. However, using electrophysiological techniques to confirm synaptic connections between neurons does come with some notable challenges. The execution of these experiments demands a high degree of specialized expertise, encompassing tasks like cell clamping, the amplifier, and fine-tuning the data acquisition system, as well as intricate data analysis. Novices might encounter a learning curve in this regard. At the same time, it is essential for the laboratory to be equipped with costly instruments and the necessary facilities. Furthermore, the patch-clamp technique must be performed after using viral tools to find potential upstream nuclei as a means to further verify synaptic connections.

Although both methods described above can be used to confirm monosynaptic connections, their prerequisites and underlying functions are different. Specifically, the modified rabies virus is employed to survey potential upstream nuclei throughout the entire brain during the initial stages of the experiment-a function absent in electrophysiological techniques. In contrast, patch-clamp technology enables a detailed analysis of monosynaptic connections by manipulating drug components in the recording system, a capability not achievable with the modified rabies virus.

## Material, Reagents, and Equipment

**Table:** 

Material	Source	Identifier
*Viral Vectors*		
RV-EnVA-ΔG-DsRed	BrainVTA	cat. # R0002
AAV2/9-rAAV-EF1α-DIO-oRVG-WPRE-hGH pA	BrainVTA	cat. # PT-0023
AAV2/9-rAAV-EF1α-DIO-H2B-EGFP-T2A-TVA-WPRE-hGH pA	BrainVTA	cat. # PT-0021
AAV2/9-hEF1α-DIO-hChR2(H134R)-EYFP-WPRE-pA	Shanghai Taitool Bioscience	cat. # S0199-9
pAAV2/9-CaMKIIα-mCherry	SunBio	cat. # PMT191
*Others*		
Tissue adhesive	3M	cat. # 1469SB
Optimal cutting temperature compound	Sakura	cat. # 4583
Borosilicate glass pipettes	Sutter Instrument	cat. # B120-69-15

Reagents	Source	Identifier
NaCl	Sigma-Aldrich, St Louis, MO, USA	cat. # S3014-1KG
KCl	Sigma-Aldrich	cat. # P5405-500G
MgSO_4_	Sigma-Aldrich	cat. # M1880-500G
NaHCO_3_	Sigma-Aldrich	cat. # S5761-1KG
KH_2_PO_4_	Sigma-Aldrich	cat. # P5655-1KG
CaCl_2_	Sigma-Aldrich	cat. # C3881-500G
MgCl_2_	Sigma-Aldrich	cat. # M0250-500G
d-glucose	Sigma-Aldrich	cat. # G7021-1KG
CsCl	Sigma-Aldrich	cat. # 289329-25G
EGTA	Sigma-Aldrich	cat. # E4378-100G
NMDG	Sigma-Aldrich	cat. # M2004-1KG
NaH_2_PO_4_	Sigma-Aldrich	cat. # 71505-1KG
HEPES	Sigma-Aldrich	cat. # H4034-1KG
Thiourea	Sigma-Aldrich	cat. # T8656-100G
l-Na-ascorbate	Sigma-Aldrich	cat. # V900326-500G
Na-pyruvate	Sigma-Aldrich	cat. # P2256
HCl	Sigma-Aldrich	cat. # H1758
Glucose	Sigma-Aldrich	cat. # G8270
QX-314	Sigma-Aldrich	cat. # 552233
ATP-Na	Sigma-Aldrich	cat. # A6419
GTP-Na	Sigma-Aldrich	cat. # 51120
4-aminopyridine (4-AP)	Sigma-Aldrich	cat. # 275875
Paraformaldehyde (PFA)	Sigma-Aldrich	cat. # 16005-1KG-R
NaH_2_PO_4_·2H_2_O	Sinopharm Chemical Reagent, Shanghai, China	cat. # 20040718
Na_2_HPO_4_·12H_2_O	Sinopharm Chemical Reagent	cat. # 10020318
Sucrose	Sinopharm Chemical Reagent	cat. # 10021418
DAPI	Beyotime	cat. # 1006
Tetrodotoxin (TTX)	Hello Bio	cat. # HB1034

Solutions	Preparation
Saline	This solution was prepared by dissolving 9 g of NaCl in distilled water for a total volume of 1 L
0.1 mol/L Phosphate buffered saline (PBS)^#^	This solution was prepared by dissolving 71.64 g of Na_2_HPO_4_·12H_2_O in distilled water for a total volume of 1 L as buffer A, dissolving 31.21 g of NaH_2_PO_4_·2H_2_O in distilled water for a total volume of 1 L as buffer B, mixing 81 mL of buffer A and 19 mL of buffer B, and adjusting osmolarity with NaCl
4% Paraformaldehyde (4% PFA)	This solution was prepared by dissolving 40 g of PFA in 0.1 M PBS for a total volume of 1 L
30% Sucrose (wt/vol)	This solution was prepared by dissolving 30 g of sucrose in 0.1 M PBS for a total volume of 1 L
Artificial cerebrospinal fluid (ACSF, cutting solution)*^#^	This solution contained (in mmol/L) 50 sucrose, 125 NaCl, 25 NaHCO_3_, 2.5 KCl, 1.25 NaH_2_PO_4_, 0.1 CaCl_2_, 4.9 MgCl_2_, and 2.5 glucose and was bubbled with carbogen (95% O_2_/5% CO_2_) (pH 7.35; 270–285 mOsm)
Recording solution*^#^	This solution contained (in mM) 125 NaCl, 25 NaHCO_3_, 2.5 KCl, 1.25 NaH_2_PO_4_, 11 glucose, 1.3 MgCl_2_ and 2.5 CaCl_2_ (pH 7.35; 270–285 mOsm)
Pipette internal solution (EPSC)*^#^	This solution contained (in mmol/L) 117 CsCH_3_SO_3_, 20 HEPES, 0.4 EGTA, 2.8 NaCl, 5 TEA, 4 MgATP, 0.3 NaGTP, 5 QX314 and 0.1 spermine at pH 7.35 (270–285 mOsm)
Pipette internal solution (IPSC)*^#^	This solution contained (in mmol/L) 130 CsCl, 1 EGTA, 10 HEPES, 2 MgATP, 0.2 NaGTP, pH 7.35 (270–285 mOsm)
*The osmotic pressure of these solutions was adjusted by a freezing point osmometer (Löser, Berlin, Germany). ^#^The pH of these solutions was adjusted by a pH meter (Mettler Toledo, Greifensee, Schweiz).	

Equipment and Software	Source	Identifier
*Equipment*		
Stereotaxic apparatus	RWD	68046
Binocular stereotaxic microscope	Olympus	SZ61
Single syringe infuse/withdraw system	KD Scientific	LEGATO 130
Micromotor Strong 90	Saeshin Precision	Strong90
Drill bits	Hager & Meisinger GmbH	B61984
Cryostat	Thermo	CryoStar NX50 OP
Olympus virtual digital slide slice scanning system	Olympus	VS120
Olympus FV-1200 laser scanning confocal microscope	Olympus	FV-1200
Vibrating blade microtome	Leica	VT1200S
Freezing point osmometer	Löser	OM-806
Digidata 1440A digitizer	Molecular Devices	Digidata 1440A
MultiClamp 700A amplifier	Molecular Devices	Multiclamp 700A
Fixed-stage upright microscope	Olympus	BX51WI
Automatic temperature controller	Warner	TC-324C
X-Cite 110LED Illumination System	Excelitas Technologies	110LED
Sutter MPC-200 micromanipulator	Sutter Instrument	MPC-200
Horizontal pipette puller	Sutter Instrument	P97
Peristaltic pump	Longer Pump	YZ1515x
Antivibration table	TMC	63-561
High Performance CCD Camera	COHU	4912-5010
Black and white monitor	Sunspot	SP-712
Precision balances	Sartorius, Göttingen, Germany	BSA822-CW
pH meter	Mettler Toledo	FE28
*Software*		
pCLAMP 10.7 software	Molecular Devices	http://www.moleculardevices.com/products/software/pclamp.html; RRID: SCR_011323
Mini Analysis Program	Synaptosoft	http://www.synaptosoft.com/MiniAnalysis/; RRID:SCR_002184
ImageJ	National Institutes of Health	https://imagej.nih.gov/ij/index.html; RRID:SCR_003070
OlyVIA	Olympus	https://www.olympus-lifescience.com/en/support/downloads/#!dlOpen=%23detail847249644; RRID:SCR_016167
FV10-ASW software	Olympus	http://www.photonics.com/Product.aspx?PRID=47380; RRID:SCR_014215
The biosafety level of laboratory when applying tracing virus is ABSL-2 (P2) qualified.		
